# *Yarrowia lipolytica* as a promising cell factory for microbial production of value-added nutraceuticals 

**DOI:** 10.3389/fbioe.2025.1673169

**Published:** 2025-08-28

**Authors:** Soseon Lee, Ju Hyeon Lee, Hyun June Park, Seung-Ho Baek

**Affiliations:** ^1^ Center for Bio-based Chemistry, Korea Research Institute of Chemical Technology (KRICT), Ulsan, Republic of Korea; ^2^ Department of Biotechnology, Duksung Women’s University, Seoul, Republic of Korea

**Keywords:** nutraceutical, yarrowia lipolytica, microbial cell factory, sphingolipid, terpenoid, flavonoid

## Abstract

The oleaginous yeast *Yarrowia lipolytica* has emerged as a powerful chassis for the sustainable production of high-value nutraceuticals. Its innate metabolism, characterized by a high flux towards the key precursor acetyl-CoA, makes it an ideal host for synthesizing complex molecules like carotenoids, flavonoids, and specialty lipids. This review summarizes recent progress in engineering *Y. lipolytica* cell factories, focusing on the synergistic application of metabolic engineering and synthetic biology. Key strategies discussed include enhancing precursor supply, redirecting metabolic flux away from competing pathways, and optimizing heterologous gene expression. We highlight the use of advanced tools like organelle compartmentalization to improve reaction efficiency and biosensor-driven screening to accelerate strain development. Furthermore, systems biology approaches utilizing multi-omics data are proving crucial for identifying novel engineering targets and overcoming metabolic bottlenecks. This review consolidates these advancements and discusses future perspectives for creating robust, industrially-relevant *Y. lipolytica* platforms for the bio-based economy.

## 1 Introduction

The global nutraceutical market has undergone significant expansion, driven by growing consumer concerns about health and wellness. However, traditional production methods, relying on direct plant extraction or complex chemical synthesis, face several challenges including low yield, seasonal availability, environmental concerns, and scalability limitations. Microbial biosynthesis has emerged as a promising alternative for sustainable nutraceutical production, offering advantages such as controlled production conditions and reduced environmental impact (Yuan and Alper, 2019; Thakur et al., 2023).

Among various microbial platforms, *Yarrowia lipolytica* has been receiving remarkable attention as a versatile platform for nutraceutical production (Zhang T. L. et al., 2023). While traditional model microbial hosts like *Escherichia coli* and *Saccharomyces cerevisiae* are well-established for biotechnological production, *Y. lipolytica* offers unique advantages for nutraceutical synthesis. Unlike *S. cerevisiae*, the intrinsic ability of *Y. lipolytica* to accumulate large amounts of lipids provides a vast intracellular pool of the key metabolic precursor acetyl-CoA, which is a critical building block for a wide array of high-value nutraceuticals, including terpenoid, flavonoid, and sphingolipid (Sun et al., 2023). The Generally Recognized as Safe (GRAS) status of *Y. lipolytica* confers significant regulatory advantages for nutraceutical production, particularly when compared to *E. coli*, which lacks GRAS designation for food-related applications. Furthermore, *Y. lipolytica* exhibits exceptional metabolic flexibility and possesses sophisticated genetic engineering tools, including CRISPR-Cas9 gene editing platforms (Lee et al., 2024). These advances have facilitated the engineering of metabolic pathways for strain improvement (Park and Ledesma-Amaro, 2023). While previous reviews have provided valuable insights into its capacity, there is growing interest in the high-value nutraceuticals that offer therapeutic potential and commercial opportunities (Madhavan et al., 2023).

This review provides a comprehensive overview of *Y. lipolytica*’s remarkable capabilities in nutraceutical production. It focuses on acetyl-CoA–derived nutraceuticals such as terpenoids, flavonoids, and sphingolipids, excepting other classes such as polyunsaturated fatty acids, phenolics, and amino acid derivatives. By consolidating recent advancements and future research directions, we identify opportunities to translate academic potential of *Y. lipolytica* into robust industrial applications for nutraceutical production.

## 2 Advances in strategies for cell factory development

### 2.1 Metabolic engineering approach

The development of efficient *Y. lipolytica*-based cell factories relies on strategic metabolic engineering to redirect cellular resources toward target product biosynthesis. Central to this approach is the manipulation of key metabolic fluxes, particularly the acetyl-CoA pool, which serves as the primary building block for numerous nutraceutical compounds (Huang et al., 2018; Marella et al., 2018).

Several strategies have been employed to enhance acetyl-CoA availability, including engineering the lipolysis pathway and the acetyl-CoA biosynthetic pathway. Enhanced lipolysis is a direct approach to increase acetyl-CoA availability. Improvement of lipolysis critically depends on the upregulation of lipase expression, whether native or heterologous, along with the optimization of lipase secretion pathways (Fickers et al., 2011; Park and Nicaud, 2020). The β-oxidation pathway, converting fatty acids to acetyl-CoA units, is a prime target for metabolic engineering associated with acetyl-CoA pool. Peroxisomal β-oxidation involves a complex enzymatic machinery including six acyl-CoA oxidases, the bifunctional enzyme, and thiolase. Coordinated overexpression of these enzymes has been shown to substantially enhance β-oxidation capacity (Liu et al., 2022; Dong T. et al., 2025). Additionally, optimization of initial step of fatty acid activation can significantly enhance acetyl-CoA production from β-oxidation (Qin et al., 2025; Yang et al., 2025). Pyruvate dehydrogenase complex (Pdc), representing a major source of acetyl-CoA from glycolytic flux, serves as a critical control point for acetyl-CoA biosynthesis. Balanced expression of subunits (Pda1, Pdb1, and Lat1) can increase the overall capacity of this multienzyme complex (Zhou et al., 2012; Guo et al., 2014). Engineering of Pdc regulation to reduce feedback inhibition is also a key strategy for sustained acetyl-CoA production (Moreira et al., 2021). Optimization of NAD^+^ regeneration systems is crucial to ensure the continued operation of the Pdc under high-flux conditions (Liu et al., 2019).

Beyond the conventional pyruvate dehydrogenase route, alternative pathways for acetyl-CoA synthesis can be engineered to provide additional flux capacity. The citrate lyase pathway represents an attractive alternative, particularly for cytosolic acetyl-CoA production (Blazeck et al., 2014; Dulermo et al., 2015). Heterologous expression of ATP citrate lyase enables the conversion of citrate to acetyl-CoA and oxaloacetate, effectively transporting acetyl units from the mitochondria to the cytoplasm. Engineering of citrate transport systems and balancing of citrate cycle flux are also essential for this strategy (Zhang et al., 2014). Overexpression of acetyl-CoA carboxylase has been shown to increase malonyl-CoA pools, essential for fatty acid synthesis and subsequent lipid-derived compounds (Tai and Stephanopoulos, 2013; Qiao et al., 2015). In addition, introduction of heterologous acetyl-CoA synthetase variants has further enhanced acetyl-CoA availability (Xu et al., 2016; Liu et al., 2019). The acetyl-CoA synthetase can convert acetate directly to acetyl-CoA in an ATP-dependent manner. Engineering of acetate availability through controlled hydrolysis of acetyl-containing compounds and optimization of ATP supply can enhance contribution of this pathway to the acetyl-CoA pool (Ma et al., 2024).

Successful cell factory development requires precise control of metabolic flux distribution. Metabolic flux redirection is the process of converting the secured precursor into the target compound without loss. This is accomplished by finely tuning the expression levels of the enzymes that catalyze each step of the biosynthetic pathway. For instance, disruption of the β-oxidation pathway prevents fatty acid degradation, thereby preserving lipid precursors for nutraceutical production (Beopoulos et al., 2008; Ledesma-Amaro et al., 2016; Ledesma-Amaro and Nicaud, 2016). Additionally, balanced expression of pentose phosphate pathway (PPP) enzymes maintains adequate NADPH supply while preserving glycolytic flux for acetyl-CoA production (Beopoulos et al., 2009; Qiao et al., 2017; Wang et al., 2020).

### 2.2 Synthetic biology-based approach

Synthetic biology is an innovative field that designs and assembles novel genetic parts and devices to build new biological systems with functions not found in nature. It provides a powerful toolbox that elevates the predictability and efficiency of metabolic engineering beyond traditional methods, enabling the creation of sophisticated cellular systems. In *Y. lipolytica*, two prominent strategies are subcellular compartmentalization and the implementation of biosensors for dynamic metabolic control (Kulagina et al., 2021; Zhang T. L. et al., 2023).

The complex cellular organelle structure of *Y. lipolytica*, including peroxisomes and lipid droplets, offers particular opportunities for metabolic compartmentalization (Navarro-Espíndola et al., 2020). This strategy involves targeting biosynthetic pathways to specific organelles to increase substrate and enzyme concentration, isolate metabolic intermediates, and alleviate the cytotoxicity (Gu et al., 2023; Ma et al., 2024). The highly developed peroxisomal system, in particular, has been exploited (Dusseaux et al., 2020; Kulagina et al., 2021). For instance, engineering peroxisomal import mechanisms through peroxisomal targeting signal modifications has enabled the successful compartmentalization of carotenoid biosynthetic pathways, resulting in improved yields (Chen et al., 2023; Park K. et al., 2024; Soldat et al., 2024). Similarly, mitochondrial engineering has shown great potential. Targeting the mevalonate (MVA) pathway to mitochondria has been demonstrated to enhance the availability of target precursor while maintaining cellular energy homeostasis (Zhu et al., 2022; Liu L. et al., 2024).

Furthermore, the implementation of biosensors enables not only high-throughput screening (HTS) for the rapid selection of high-efficiency strains, but also dynamic real-time control of metabolic pathways (Qiu et al., 2020; Arnesen and Borodina, 2022). Transcription factor-based biosensors that respond to key metabolites such as acetyl-CoA, malonyl-CoA, and farnesyl diphosphate have been successfully developed (Wei et al., 2017; Liu S. C. et al., 2024). These biosensors can be integrated into feedback control circuits that automatically regulate gene expression in response to the intracellular concentration of a target molecule, to optimize metabolic flux and product formation.

### 2.3 Systems biology-based approach

To overcome the limitations of conventional metabolic engineering, systems biology-based approaches are increasingly being adopted for the rational development of *Y. lipolytica* cell factories. The application of systems biology through comprehensive multi-omics analysis has provided a powerful tool for identifying the engineering targets. In particular, genome-scale metabolic models (GEMs) integrated with transcriptomic, proteomic, and metabolomic data provide unprecedented insights into cellular behavior and bottlenecks (Kavscek et al., 2015; Wei et al., 2017; Yang et al., 2024).

This approach has proven highly effective across different classes of nutraceuticals. In terpenoid production, for example, comparative transcriptomics has revealed key competing pathways, enabling targeted gene deletions that significantly boost precursor flux (Worland et al., 2020; Zhang et al., 2020). Similarly, for bioactive lipids, a multi-omics analysis identified a critical link between amino acid catabolism and product synthesis, leading to gram-scale titers (Ma et al., 2020; Gasovic et al., 2023). In polyphenol biosynthesis, systems-level optimization of heterologous gene expression has been critical for balancing complex pathways and minimizing byproduct formation (Wang et al., 2022; Mitri et al., 2024). Overall, the shift towards data-driven, systems-level engineering is accelerating the development of robust strains for high-value nutraceutical production.

## 3 Nutraceutical production in *Y. lipolytica* as cell factory

### 3.1 Terpenoids production

**FIGURE 1:**
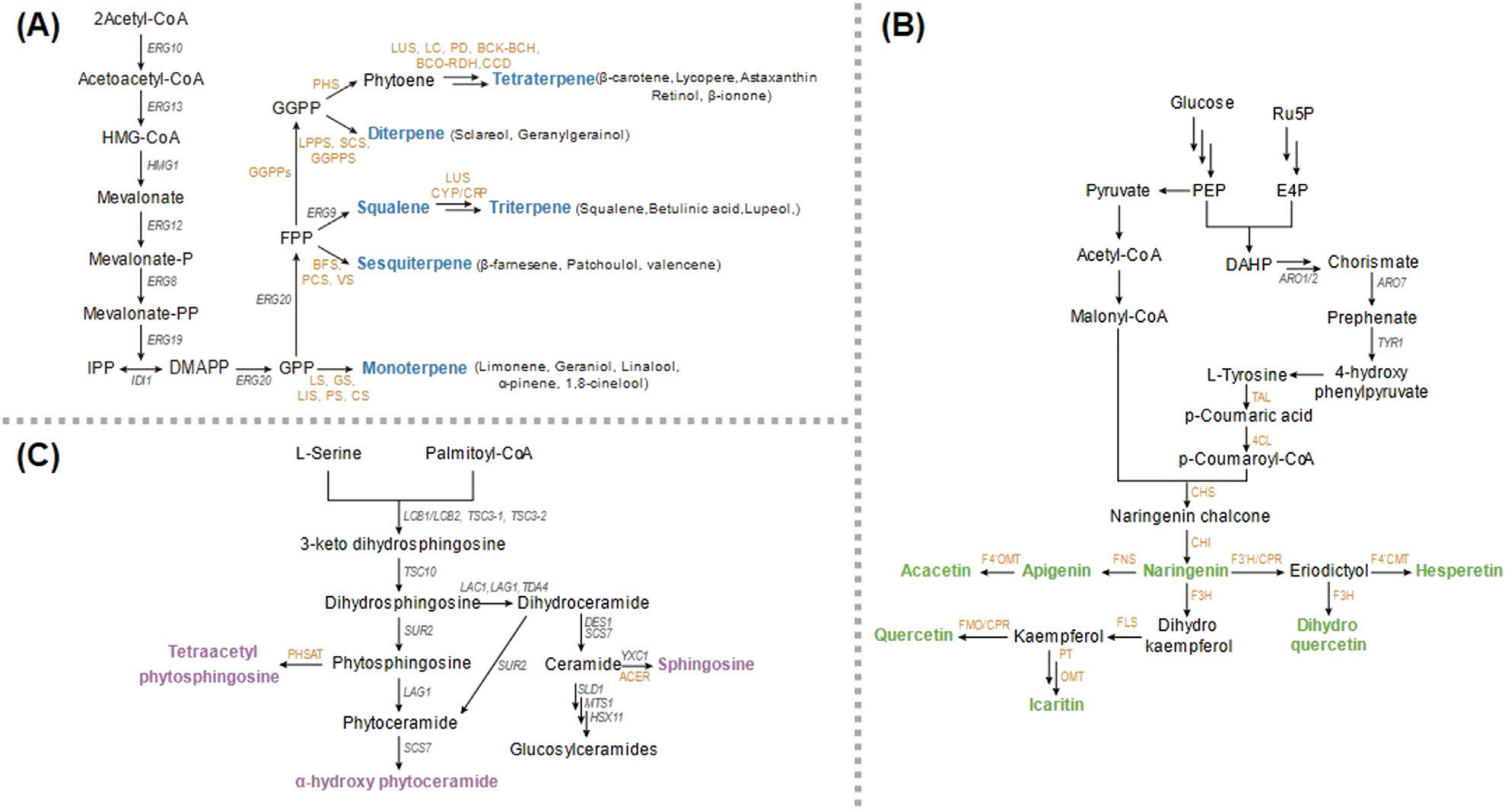
Biosynthetic pathways of major nutraceuticals in *Y. lipolytica*. **(A)** Terpenoid biosynthesis: The pathway starts from acetyl-CoA and proceeds through the mevalonate (MVA) pathway to produce IPP and DMAPP, the universal C5 precursors. **(B)** Flavonoid biosynthesis: This pathway utilizes precursors from both the shikimate pathway and acetyl-CoA pool. **(C)** Sphingolipid biosynthesis: This pathway begins with serine and palmitoyl-CoA to form the basic ceramide structure. Metabolites: HMG-CoA, 3-hydroxy-3-methylglutaryl-CoA; IPP, isopentenyl pyrophosphate; DMAPP, dimethylallyl pyrophosphate; E4P, erythrose 4-phosphate; PEP, 2-phosphoenolpyruvate; Ru5P, ribulose 5-phosphate; DAHP, 3-deoxy-D-arabinoheptulosonate 7-phosphate. Genes and Enzymes: ERG10, acetyl-CoA acetyltransferase; ERG13, 3-hydroxy-3-methylglutarylCoA synthase; HMG1, 3-hydroxy-3-methylglutarylCoA reductase; ERG12, mevalonate kinase; ERG8, phosphomevalonate kinase; ERG19, mevalonate diphosphate decarboxylase; IDI1, isopentenyl diphosphate isomerase; ERG20, geranyl/farnesyl diphosphate synthase; ERG9, squalene synthase; PS, α-pinene synthase; CS, 1,8-cinelool synthase; BFS, β-farnesene synthase; PD, phytoene desaturase; BCO, β-carotene 15,15′-dioxygenase; RDH, retinol dehydrogenase; CCD, carotenoid-cleaving dioxygenase; ARO1, ARO2, multifunctional enzymes; ARO7, chorismite mutase; TYR1, prephenate dehydrogenase; TAL, tyrosine ammonia lyase; 4CL, 4-coumaroyl-CoA ligase; CHS, chalcone synthase; CHI, chalcone isomerase; FNS, flavone synthase; F4′OMT, flavonoid 4′-O-methyltransferase; F3′H, flavonoid 3′-hydroxylase; CPR, cytochrome P450 reductase; F3H, flavanone-3-hydroxylase; FLS, flavanol synthase; FMO, flavonoid 3′-monooxygenase; PT, prenyltransferase; OMT, O-methyltransferase; LCB1, LCB2, TSC3-1, TSC3-2, serine palmitoyltransferase; TSC10, 3-ketosphinanine reductase; LAC1, LAG1, TDA4, ceramide synthase; SUR2, c4-hydroxylase; SCS7, sphingolipid α-hydroxylase; DES1, sphingolipid Δ4-desaturase; YXC1, ceramidase; SLD1, sphingolipid Δ8-desaturase; MTS1, sphingolipid C9 methyltransferase; HSX11, Glucosylceramide synthase; PHSAT, phytosphingosine acetyl transferase; ACER, alkaline ceramidase.

The oleaginous nature of *Y. lipolytica* provides intrinsic advantages for terpenoids biosynthesis, owing to its efficient lipid metabolism, which can be redirected through targeted metabolic engineering (Figure 1A). Significant progress has been made in the production of various terpenoids (Table 1).

**TABLE 1:** Engineering of *Y. lipolytica* for various neutraceutical production.

Product	Strain	Main strategies	Titer (scale)	References
Terpenoids				
Monoterpenoid				
Limonene	Po1d	Overexpressing *HMGR*, *ERG20*, and limonene synthase from *Citrus limon*; Peroxosimal compartmentalization	0.069 g/L (bioreactor)	Park Y. K. et al. (2024)
Linalool	W29	Overexpressing *ERG20* ^*F88W−N119W*^, linalool synthase from *Actinidia argute*, and *GPPS* from *Catharanthus roseus*; *dgk1Δ*	0.109 g/L (flask)	Taratynova et al. (2024)
Geraniol	Po1f	Overexpressing *tHMG1*, *IDI1*, and geraniol synthase from *Catharanthus roseus*	1 g/L (flask)	Agrawal et al. (2023)
Sesquiterpenoid				
β-farnesene	Po1f	Overexpressing β-farnesene synthase from *Artemisia annua*, malic enzyme from *Mucor circinelloides*, and ATP-citrate lyase from *Mus musculus*	28.9 g/L (bioreactor)	Bi et al. (2023)
Patchoulol	Po1f	Overexpressing *tHMG1* and patchoulol synthase from *Pogostemon cablin; ERG9 p*romoter replacement	2.864 g/L (bioreactor)	Peng et al. (2023)
Valencene	W29	Overexpressing fused *ERG8*-*ERG19*, and *ERG20*-valencene synthase mutant from *Eryngium glaciale*	3.34 g/L (bioreactor)	Chen Y. et al. (2025)
Diterpenoid				
Sclareol	Po1f	Overexpressing *tHMG1* with truncated LPP synthase and sclareol synthase from *Salvia sclarea*	12.9 g/L (bioreactor)	Sun et al. (2024)
Geranylgeraniol	Po1f	Overexpressing *tHMG1*, *ERG20, DPP1* from *Saccharomyces cerevisiae*, and GGPP synthase from *Pantoea agglomerans*	3.346 g/L (flask)	Wang et al. (2024)
Triterpenoid				
Squalene	Po1f	Overexpressing *tHMG1*, *IDI1,* and *ERG9*; Peroxosimal compartmentalization	51.2 g/L (bioreactor)	Ning et al. (2024)
Tetraterpenoid				
β-carotene	Po1f	Overexpressing *tHMG1, ERG12, IDI1, and ERG20* with CarRP^Y27R^ and CarB from *Mucor circinelloides*; Introducing synthetic isopentenol utilization pathway	39.5 g/L (bioreactor)	Ma et al. (2022)
Lycopene	Po1f	Overexpressing *HMG1*, *GGS1*, and *ERG12*; Multiple integration of *crtE*, *crtB*, and *crtI* from *Lamprocystis purpurea*	5.1 g/L (bioreactor)	Luo et al. (2023)
Sphingolipids				
Non-acetylated Dihydrosphingosine	Po1f	Disrupting *SUR2* and *SLD1*; Overexpressing alkaline ceramidase from mouse	1.4 g/L (bioreactor)	Shin et al. (2025)
Tetraacetyl phytosphingosine	Po1f	Overexpressing acetyl transferases from *Wickerhamomyces ciferrii*; Disrupting *LCB4*	0.65 g/L (bioreactor)	Han et al. (2021)
Flavonoids				
Naringenin	Po1f	Multiple integration of naringenin biosynthetic pathway	8.3 g/L (bioreactor)	Liu M. S. et al. (2024)
Acacetine	Po1f	Overexpressing *TKL1* with heterologous flavone synthase and flavonoid 4′-O-methyltransferase; Downregulating *FAS1*	1.1 g/L (flask)	Park et al. (2025)
Dihydroquercetin	Po1f	Overexpressing flavone-3-hydroxylase from *Solanum lycopersicum* and *Vitreoscilla* hemoglobin	4.2 g/L (bioreactor)	He et al. (2025)

Recent advancements in monoterpenoid production have primarily involved the heterologous expression of plant-derived monoterpene synthases (MS). Numerous efforts have focused on enhancing acetyl-CoA supply and optimizing the expression of key enzymes in the MVA pathway, such as HMG-CoA synthase and reductase. D-limonene production, for example, has been demonstrated through expressing a fused construct of Erg20 and *Citrus limon* D-limonene synthase (LS) along with HMG-CoA reductase, yielding up to 69.3 mg/L (Park Y. K. et al., 2024). Linalool has been produced via heterologous expression of (S)-linalool synthase (LIS) and geranyl pyrophosphate (GPP) synthase (Taratynova et al., 2024). Geraniol titers of approximately 1 g/L in flask fermentation were achieved through overexpressing of three copies of a plant-derived geraniol synthase (GS), along with single-copy of truncated *HMG1* (*tHMG1*), *IDI1*, *ERG10*, and *ERG13* (Agrawal et al., 2023). Additional monoterpenes, such as α-pinene and 1,8-cinelool, have been produced by expressing their respective synthases (Wei et al., 2021; Bhoir et al., 2025).

Sesquiterpenoid production has targeted high-value compounds such as β-farnesene, patchoulol, and valencene. By introducing heterologous malic enzyme and ATP-citrate lyase, coupled with fermentation process optimization, β-farnesene production titers reached 28.9 g/L (Bi et al., 2023). Patchoulol biosynthesis was enhanced by integrating a mutant patchoulol synthase (PCS) from *Pogostemon cablin* and optimizing farnesyl pyrophosphate (FPP) availability, resulting in a 1684-fold increase and final titers of 2.864 g/L in bioreactor fermentation (Peng et al., 2023). Likewise, valencene production has been achieved titers of up to 3.34 g/L by screening and selection of highly active valencene synthase (VS) (Chen Y. et al., 2025).

Diterpenoid production represents greater challenges due to the complexity of geranylgeranyl diphosphate (GGPP) biosynthesis and the requirement for specialized enzymes. Sclareol production has been demonstrated through GGPP accumulation, enabling by MVA pathway optimization and co-expression of (13E)-8α-hydroxylabden-15-yl diphosphate synthase (LPPS) and sclareol synthase (SCS) from *Salvia sclarea* (Sun et al., 2024; Chen J. et al., 2025). Geranylgeraniol, an important component in essential oils, has reached 3.346 g/L in the flask fermentation via overexpression of GGPP synthase (GGPPS) and a heterologous GGPP phosphatase (GGPPP) (Wang et al., 2024).

Triterpenoid production has focused on bioactive compounds. The production of squalene, a representative high-value acyclic triterpenoid compound, has exceeded 51.2 g/L by combining strategies including multi-copy gene integration, peroxisomal compartmentalization, and enhanced FPP availability (Liu Z. Y. et al., 2024; Ning et al., 2024). The biosynthesis of lupeol and betulinic acid has been achieved through expression of plant-derived lupeol synthases (LS) and cytochrome P450 monooxygenases (CYP) with cytochrome P450 reductase (CRP), along with engineering of the MVA pathway and lipid metabolism (Jin et al., 2019; Zhang et al., 2020; Li et al., 2025). However, these efforts are still constrained by the complex enzymatic machinery and cofactor requirements.

Among terpenoid classes, tetraterpenoids, particularly carotenoids, represents one of the most successful terpenoids produced in *Y. lipolytica*. β-carotene production has reached over 39.5 g/L through the reconstruction of the carotenoid biosynthesis pathway using phytoene synthase (PHS) and lycopene cyclase (LC) from *Xanthophyllomyces dendrorhous* (CrtI, and CrtYB) and *Mucor circinelloides* (CarB, and CarRP). This achievement was driven by optimized codon usage, balanced enzyme expression, and protein engineering to alleviate substrate inhibition (Liu et al., 2021; Ma et al., 2022). Lycopene production has been improved to over 5.1 g/L through multicopy integration of bacterial *crtE*, *crtB*, and *crtI* genes from *Pantoea ananatis* (Luo et al., 2023). Astaxanthin, a high-value carotenoid with pharmaceutical applications, has been produced at up to 2.8 g/L through the complete pathway implementation, including β-carotene hydroxylase (BCH) and ketolase (BCK) enzymes from algae (Abdullah et al., 2025). Additionally, production of zeaxanthin and β-cryptoxanthin has been demonstrated through pathway optimization (Zhang G. L. et al., 2023). Other C40 terpenoids such as retinol and β-ionone have also been produced through engineered biosynthetic pathways (Chen et al., 2022; Park et al., 2022; Ren et al., 2024; Shi et al., 2024).

### 3.2 Sphingolipids production

Sphingolipids are essential structural components of eukaryotic plasma membranes and play a crucial role in cellular processes such as cell signaling. Sphingolipid biosynthesis in *Y. lipolytica* encompasses diverse bioactive compounds with significant nutraceutical potential because this microorganism possesses native C4-desaturase gene for synthesis of sphingosine-based sphingolipids (Murakami et al., 2015; Megyeri et al., 2016; Shin et al., 2025). The foundation of sphingolipid production relies on sphingoid bases, particularly sphingosine, dihydrosphingosine, and phytosphingosine (Figure 1C).

Ceramide production represents a challenging endeavor requiring coordinated expression of multiple enzymes including serine palmitoyltransferase, 3-ketodihydrosphingosine reductase, and ceramide synthase. Recent research showed a new possibility for human glucosylceremides production in *Y. lipolytica* via disrupting C4-hydroxylase and Δ8 desaturase. In addition, integration of heterologous alkaline ceramidase is an important strategy to enhance production levels of non-acetylated long-chain bases, including dihydrosphingosine and sphingosine (Shin et al., 2025).

Tetraacetyl-phytosphingosine, an acetylated derivative of phytosphingosine, has been produced at up to 650 mg/L via co-expression of *Wickerhamomyces ciferrii* derived acetyl transferases, Sli1p and Atf2p and sphingoid long-chain base kinase gene deletion, along with optimized culture conditions (Han et al., 2021).

### 3.3 Flavonoids production

Recent progress has established *Y. lipolytica* as a promising chassis for flavonoids biosynthesis, with initial efforts has focused on the efficient production of the key precursor naringenin (Figure 1B). This was achieved by enhancing shikimate flux and increasing the availability of malonyl-CoA and erythrose-4-phosphate. Major engineering strategies included the overexpression of enzymes such as Aro4^K221L^, Aro2, Aro7^G139S^, a heterologous tyrosine ammonia lyase, and transketolase, along with downregulation of fatty acid synthase and 4-hydroxyphenylpyruvate dioxygenase (Park et al., 2025). Utilizing a high-efficiency multi-copy integration system, naringenin production was increased to 8.3 g/L in fed-batch fermentation (Liu M. S. et al., 2024).

Based on naringenin-producing strain, acacetin production has been accomplished at a titer of 1.1 g/L by introducing codon-optimized flavone synthase and flavonoid 4′-O-methyltransferase. Enzyme copy number optimization and controlled fermentation conditions further enhanced the production yield (Park et al., 2025). A similar modular engineering strategy was applied to produce dihydroquercetin. By identifying and amplifying a highly active flavanone-3-hydroxylase, and improving cofactor supply and oxygen availability, dihydroquercetin production has reached 4.2 g/L in fed-batch culture (He et al., 2025). In addition, other flavonoids, including quercetin, icaritin, and hesperetin have been successfully produced in *Y. lipolytica* through various engineered biosynthetic pathways (Dong Y. X. et al., 2025; Sun et al., 2025; Wang et al., 2025).

## 4 Conclusion and future perspectives

*Y. lipolytica* has established as a versatile platform for nutraceutical production, demonstrating biosynthetic capabilities across diverse compounds (Table 1). The achievement of high-value products such as β-carotene and ceramides emphasizes its significant potential for sustainable nutraceutical production (Shin et al., 2025).

Despite these promising developments, several challenges remain, including the need to achieve economically viable production levels for complex nutraceuticals while managing inherent metabolic trade-offs (Lee et al., 2025; Shin et al., 2025). However, rapid progress in synthetic biology and systems biology provide possibilities for overcoming these limitations. Current metabolic engineering strategies typically focus on individual pathways without considering global metabolic networks and regulatory mechanisms. Next-generation strain development requires integrated multi-scale modeling approaches that simultaneously account for metabolic, regulatory, and evolutionary restrictions (Kim et al., 2019; Gasovic et al., 2023; Duman-Özdamar et al., 2025).

The integration of kinetic models with regulatory networks will enable more accurate prediction of engineering outcomes, while machine learning applications to large-scale omics datasets can reveal hidden regulatory relationships and guide sophisticated control strategies. The combination of rational metabolic engineering with evolutionary approaches, particularly adaptive laboratory evolution (ALE) provides synergistic opportunities for robust strain development (Chen et al., 2024; Yook et al., 2025). ALE under production-relevant conditions can identify beneficial mutations inaccessible through rational design alone, and its integration with systematic genome editing can accelerate strain optimization.

CRISPR-based technologies beyond conventional gene editing offer opportunities for precise metabolic control (Schwartz et al., 2019). CRISPR interference (CRISPRi) and CRISPR activation (CRISPRa) systems enable reversible, tunable gene regulation without permanent modifications (Schwartz et al., 2018; Misa and Schwartz, 2021). Multiplexed CRISPR platforms allow simultaneous modulation of multiple targets, enhancing the precision and complexity of metabolic engineering strategies (Gao et al., 2016; Bae et al., 2020).

Synthetic biology facilitates the construction of sophisticated regulatory circuits, including biosensors for real-time monitoring and dynamic flux control. Improving heterologous enzyme performance through directed evolution and rational protein engineering will be crucial for enhanced productivity (Zhang and Shi, 2021; Hu et al., 2024).

Compartmentalizing biosynthetic pathways within specific organelles represents a promising strategy for reducing metabolic burden and optimizing cofactor availability (Kulagina et al., 2021). Well-developed peroxisomal and mitochondrial systems of *Y. lipolytica* offer excellent platforms for implementing this approach.

Beyond metabolic capabilities, practical considerations for industrial-scale production are critical. The high lipid content of *Y. lipolytica* can complicate the extraction and purification of the target nutraceutical from the complex biomass matrix. Therefore, developing cost-effective and efficient recovery processes is a key area of ongoing research to ensure the economic viability of *Y. lipolytica*-based production platforms.

The integration of these advanced approaches with a systems-level understanding holds significant promise for fully realizing the potential of *Y. lipolytica* in sustainable nutraceutical production. Future success will depend on continued advances in systems biology, sophisticated engineering tools, and multiple optimization strategies. Ultimately, *Y. lipolytica*-based production systems will play an increasingly crucial role for high-quality, sustainable nutraceuticals.
